# White paper: standards for handling and analyzing plant pan-genomes

**DOI:** 10.12688/f1000research.166538.1

**Published:** 2025-07-28

**Authors:** Marc C. Heuermann, Pedro Barros, Sebastian Beier, Heidrun Gundlach, Jorge Alvarez-Jarreta, Keywan Hassani-Pak, Patrick König, Anne Fiebig, Tim Godec, Kristina Gruden, Nadja Nolte, Marko Petek, Uwe Scholz, Maja Zagorščak, Klaas Vandepoele, Michiel Van Bel

**Affiliations:** 1 Leibniz Institute of Plant Genetics and Crop Plant Research (IPK), Seeland, Saxony-Anhalt, 06466, Germany; 2 Universidade Nova de Lisboa Instituto de Tecnologia Quimica e Biologica, Oeiras, Lisbon, Portugal; 3 Forschungszentrum Jülich GmbH Institute of Bio- and Geosciences, Jülich, North Rhine-Westphalia, 52425, Germany; 4 Helmholtz Zentrum München, Neuherberg, 85764, Germany; 5 Systems Immunity Research Institute, Cardiff University School of Medicine, Cardiff, Wales, UK; 6 Rothamsted Research, Harpenden, England, AL52JQ, UK; 7 National Institute of Biology, Večna pot 111, Ljubljana, 1000, Slovenia; 8 Department of Plant Biotechnology and Bioinformatics, Ghent University, Technologiepark 71, Ghent, 9052, Belgium

**Keywords:** plant pan-genome, white paper, standards, quality control

## Abstract

Plant pan-genomes, which aggregate genomic sequences and annotations from multiple individuals of a species, have emerged as transformative tools for understanding genetic diversity, adaptation, and evolutionary dynamics. Super-pan-genomes, extending across species boundaries, further enable comparative analyses of clades or genera, bridging breeding applications with evolutionary insights ( Shang et al., 2022; Li et al., 2023a). However, the absence of standardized practices for data generation, analysis, and sharing hinders reproducibility and interoperability. This white paper presents a harmonized framework developed by the ELIXIR E-PAN consortium, addressing nomenclature, quality control (QC), data formats, visualization, and community practices. By adopting these guidelines, researchers can enhance FAIR (Findable, Accessible, Interoperable, Reusable) compliance, foster collaboration, and accelerate translational applications in crop improvement and evolutionary biology.

## 1. Introduction

Pan-genomes capture both core genomic elements (shared across individuals) and accessory components (variable or unique to subsets), offering unprecedented resolution for studying traits such as disease resistance, environmental adaptation, and domestication ( Qin et al., 2021; Zhou et al., 2022). Super-pan-genomes, which span multiple species, provide evolutionary context for gene family dynamics and speciation events, as demonstrated in clades like Brassicaceae ( Jiao & Schneeberger, 2020) and Solanaceae ( Alonge et al., 2022). In plant genomics, pan-genomes are vital for understanding genetic diversity, adaptation, and evolutionary dynamics, particularly given the extensive variation observed in plant species ( Schreiber et al., 2024). Despite their potential, inconsistencies in data management—such as ad hoc naming conventions, variable QC practices, and fragmented repository use—limit cross-study comparisons and data reuse.

The ELIXIR E-PAN consortium synthesizes insights from foundational studies on barley ( *Hordeum vulgare*), rice ( *Oryza sativa*), tomato ( *Solanum lycopersicum*), and Arabidopsis ( *Arabidopsis thaliana*) to propose actionable standards. These guidelines aim to unify the plant genomics community, ensuring robust, interoperable resources for breeding and evolutionary research.

## 2. Naming conventions and ontologies

### 2.1 Accession and assembly identifiers

Accession naming should adhere to MIAPPE (Minimum Information About Plant Phenotyping Experiments) standards. The Biological Material ID should incorporate institutional identifiers, followed by the accession number from germplasm catalogue or common name of the plant source/variety (e.g., IPK-Gatersleben:HOR_13170 for barley accession “Barke”) to ensure traceability (MIAPPE v1.1, Papoutsoglou et al., 2020). When complementary data regarding a specific accession is also available at external sources (e.g. Biosamples), a link to a Biological material external ID should be provided in the metadata.

Genome assembly identifiers should contain at least 4 fields—species, variety/line, project group, assembly version — separated by period (‘.’), with an optional fifth field for additional information ( Cannon et al., 2025). For example, drOrySati.Nipponbare.RicePan.1.0, which refers to the assembly of *Oryza sativa*, Nipponbare cultivar, RicePan project, version 1.0 (ToLID identifier, https://id.tol.sanger.ac.uk/, Darwin Tree of Life Consortium, 2023).

### 2.2 Gene identifiers

Gene identifiers must balance stability with biological relevance, as outlined by Cannon et al. (2025), keeping track of the annotation version, chromosome and gene ID. Their framework proposes human- and machine-readable identifiers, including the assembly names (e.g. drOrySati.Nipponbare.RicePan.1.0) with the addition of gene models like drOrySati.Nipponbare.RicePan.1.0.1.01.g000100 (assembly version 1.0, annotation version 1, chromosome 01, gene 100). To enhance this for pan-genomics, the “group” field can denote pan-genome projects (e.g., RicePan), linking multiple assemblies, while optional fields like “Hap1” or metadata tags distinguish haplotypes or accession types (e.g., wild vs. cultivated). Pangenes, representing orthologous gene clusters, can be assigned identifiers like drOrySati.RicePan.pan00001, with metadata linking to specific gene models across assemblies. Cannon et al. (2025) advocate preserving legacy identifiers via cross-references to ensure stability, avoiding disruptive renaming as new accessions are added.

### 2.3 Metadata and ontologies

A core metadata schema is critical for interoperability. Required fields to properly annotate pan-genome studies include species details such as name (TaxonID), pedigree, geographic origin, ploidy and chromosome number, as well as sequencing technology used (e.g. PacBio HiFi, Oxford Nanopore, Hi-C, Illumina), assembly pipelines (e.g., Flye ( Kolmogorov et al., 2020), hifiasm ( Cheng et al., 2024), Canu ( Koren et al., 2017), …), and assembly QC metrics (e.g., BUSCO scores ( Manni et al., 2021)). Existing ontologies such as the Plant Ontology (PO) and Gene Ontology (GO) or probably more suited the Sequence Ontology (SO) should be extended to include pan-genome-specific terms that describe the layouts and structures of pan-genomes ( Plant Ontology Consortium, 2023, The Gene Ontology Consortium, 2023; Eilbeck et al., 2005). These can be categorized as core, shell and cloud genome genes, but these terms may depend on the number of genomes and genotypes selected ( Jayakodi et al., 2024). Collaboration with the AgBioData Nomenclature Working Group and the Genomics Standards Consortium ( https://www.gensc.org/) ensures alignment with broader genomic standards ( Cannon et al., 2025).

## 3. Quality Control (QC) standards

### 3.1 Sequencing and assembly QC

Quality control in genome assembly workflows begins with sequencing QC, where tools like FASTQC assess raw read integrity and adapter content, while k-mer plots generated via Jellyfish ( Marçais et al., 2011) paired with GenomeScope ( Ranallo-Benavidez et al., 2020) estimations reveal genome complexity, including ploidy, heterozygosity, and repetitive element profiles. For QC of individual assemblies, metrics such as contiguity, completeness, and consensus accuracy are evaluated using QUAST ( Mikheenko et al., 2023) and CRAQ ( Li et al., 2023b), with Merqury validating haplotype resolution in polyploid or heterozygous genomes (e.g., wheat, potato) by comparing k-mer spectra between raw reads and assemblies ( Rhie et al., 2020). Finally, repeat QC ensures assembly integrity via the LTR Assembly Index (LAI, Ou et al., 2018) to evaluate retrotransposon completeness and tidk ( Brown et al., 2025) for telomere motif identification, safeguarding chromosomal end-to-end accuracy.

### 3.2 Annotation QC

In addition to the assembly strategy used, the corresponding annotation pipelines should also be documented. These may include gene model integration pipelines based on MAKER2 ( Holt & Yandell, 2011), PASA ( Haas et al., 2003) or BRAKER3 ( Gabriel et al., 2024), or more advanced tools integrating deep learning methods like Helixer ( Stiehler et al., 2020) for ab initio prediction. Additionally, Liftoff ( Shumate & Salzberg, 2021) for annotation transfer should be integrated into versioned workflows (e.g., Snakemake ( Köster & Rahmann, 2012), Nextflow ( Di Tommaso et al., 2017)). Transcriptomic data (RNA-Seq) may be used to validate gene models, particularly for accessory genes lacking orthologs ( Qin et al., 2021). It is important to ensure that multiple tissues, such as roots and shoots, are represented, and that sufficient replication is included to capture transcriptome diversity.

QC of genome structural annotation may leverage lineage-specific BUSCO analyses to assess gene space completeness (adjusted for polyploidy; Manni et al., 2021), complemented by Mercator4 ( Bolger et al., 2021) for automated gene family classification, PSAURON for structural annotation validation ( Sommer et al., 2025), and OMArk for contamination detection through evolutionary consistency checks ( Nevers et al., 2025).

### 3.3 Pan-genome-specific QC

Pan-genome completeness requires saturation analysis, where gene accumulation curves determine whether new accessions yield novel genes ( Tettelin et al., 2005). For species like barley, benchmark datasets of 100+ conserved genes enable orthology tool validation ( Jayakodi et al., 2024). Additionally, gene family expansion and contraction analyses, facilitated by tools such as OrthoFinder ( Emms & Kelly, 2019) and CAFE5 ( Mendes et al., 2020), provide insights into evolutionary dynamics across accessions. Structural variant detection (e.g., using Sniffles2 ( Smolka et al., 2024) or SVIM ( Heller & Vingron, 2019)) must quantify representation of indels and inversions ( Qin et al., 2021). Similarly, presence-absence variation (PAV) detection, enabled by tools like Panaroo or PAV-specific pipelines, is critical for identifying variable gene content that contributes to phenotypic diversity ( Tonkin-Hill et al., 2020).

## 4. Data formats and sharing

### 4.1 File formats

• **Raw data**: Assemblies must be submitted in FASTA format with headers containing unique sequence identifiers (e.g., >chr1, >chr2). Annotations must be provided in GFF3 or GTF format (compliant with Sequence Ontology), with the sequence IDs in the first column exactly matching the sequence identifiers used in the FASTA headers.
• **Derived data**: Structural variants in VCF/BCF, orthogroups in TSV (cluster ID + member gene), and graph-based representations (GFA format) for complex pan-genomes ( Li et al., 2020).

### 4.2 Repositories

A centralized E-PAN repository hosted by ELIXIR will archive versioned datasets (e.g., Barley v2, Rice v1.5) with DOI-based identifiers (DataCite). Public deposition in INSDC (raw reads and assembly) and Ensembl Plants (annotations, see documentation of Ensembl, 2025) ensures global accessibility (ENA Documentation, 2025).

### 4.3 Metadata requirements

Mandatory metadata fields include sequencing technology and coverage (e.g., PacBio HiFi, Oxford Nanopore), assembly method (e.g., Flye ( Kolmogorov et al., 2020), Canu ( Koren et al., 2017)), and accession provenance (BioSample IDs). Missing metadata, as observed in early barley submissions, must be addressed via enforced submission guidelines ( Jayakodi et al., 2024).

## 5. Visualization and analysis guidelines

### 5.1 Visualization tools

Plant pan-genomes capture a species’ full genomic diversity, constructed using **linear-based** or **graph-based** methods, each with distinct strengths and limitations.

**Linear-based** approaches align genomes to a single reference or consensus sequence, identifying SNPs, indels, and presence/absence variations (PAVs) using tools like Ensembl Compara ( Dyer et al., 2025) or OrthoFinder ( Emms & Kelly, 2019). These methods are simple, computationally efficient, and compatible with tools like JBrowse2 or IGV for synteny and PAV visualization ( Diesh et al., 2023; Robinson et al., 2023). However, reference bias limits their ability to capture complex structural variations, particularly in repetitive or polyploid plant genomes.

**Graph-based** approaches model genomes as interconnected nodes (shared regions) and edges (SNPs, indels, structural variants) using tools like VG Toolkit ( Hickey et al., 2020), PGGB ( Garrison et al., 2024), PanTools ( Jonkheer et al., 2022), and wfmash ( Guarracino et al., 2021). They provide an unbiased, comprehensive view of genomic diversity, ideal for complex genomes like tomato ( Zhou et al., 2022). Visualization tools like Bandage ( Wick et al., 2015) or Cytoscape ( Shannon et al., 2003) address structural complexity but are computationally intensive and require specialized expertise.

In essence, linear methods suit rapid, basic analyses, while graph-based approaches excel for complex structural variations despite greater computational demands. As tools and computational resources advance, graph-based methods are becoming more accessible, enhancing plant pan-genome studies. A multitude of tools are summarized in Naithani et al. (2023).

### 5.2 Analysis best practices

Orthology inference tools (e.g., OrthoFinder, OMA) must be benchmarked using inflation value sweeps to minimize false positives ( Emms & Kelly, 2019). Trait association studies should integrate pan-genomes with GWAS/QTL data, as demonstrated in rice ( Qin et al., 2021), and deliver such integrated knowledge graphs to assist scientists and breeders in evidence-based gene discovery as being developed at KnetMiner ( Hassani-Pak et al., 2021).

## 6. Case studies

1. **Barley Pan-genome** ( Jayakodi et al., 2024): The IPK barley pan-genome (76 accessions) highlighted challenges in polyploid assembly and manual curation. Automated QC pipelines (Snakemake ( Köster & Rahmann, 2012)) and validation gene sets improved reproducibility.
2. **Rice Pan-genome** ( Qin et al., 2021): Analysis of 31 accessions revealed hidden structural variations using Sniffles, underscoring the need for standardized QC metrics.
3. **Tomato Super-Pan-genome** ( Zhou et al., 2022): A graph-based representation of 838 genomes resolved complex structural variants, informing breeding for disease resistance.
4. **Arabidopsis** ( Jiao & Schneeberger 2020; Zhong et al., 2025): Annotation transfer across eight high-quality genomes demonstrated the utility of Liftoff for cross-accession consistency. Multi-omic, pan-genomic assessment and comparison of Arabidopsis genomes that constitute the Arabidopsis MAGIC population.

## 7. Future directions

• **Machine learning**: Tools like DeepVariant ( Poplin et al., 2018) will enhance variant calling in polyploid genomes. Detection of other genomic features, such as repeat elements, regulatory elements, and binding sites, will be enabled and refined using foundational models, as demonstrated in recent high-impact studies. For instance, BigRNA predicts tissue-specific RNA expression and identifies regulatory elements like microRNA and protein binding sites with high accuracy ( Celaj et al., 2023). Similarly, Evo 2 detects transcription factor binding sites and exon-intron boundaries across diverse genomes ( Brixi et al., 2025), while models like DNABERT ( Ji et al., 2021) and Enformer ( Avsec et al., 2021) excel in promoter prediction and variant effect analysis ( Li et al., 2024). These advancements highlight the transformative potential of foundational models in refining genomic feature detection, particularly for complex polyploid genomes.
• **Cross-species standards**: Develop clade-wide frameworks (e.g., Brassicaceae) to unify super-pan-genome analyses.
• **Community engagement**: ELIXIR hackathons will refine workflows and ontology terms, ensuring adaptability to technological advances.
• **Generalized feature identification:** As pan-genome graphs grow to encompass not just core and variable genes but a full spectrum of genomic elements, we need a unified identification system. Current annotation often focuses on genes, leaving features like transposable elements, SSRs, non-coding RNAs, and regulatory motifs with inconsistent or tool-specific labels. We propose the development of a **generalized feature identifier (GFI)**. This system would provide a stable, queryable, and standardized format for any annotated feature, independent of its type or the discovery tool used. A GFI would be important for pan-genome-scale association studies and for functionally characterizing the entire “dark matter” of the genome, ensuring that a SNP in a long terminal repeat or a copy number variation in a novel ncRNA can be cataloged and compared with the same rigor as in a gene.

## 8. Conclusion

This white paper establishes a community-driven framework for plant pan-genome research. By adopting these guidelines, researchers can ensure data interoperability, reproducibility, and translational impact. The E-PAN consortium calls for global collaboration to iteratively refine these standards, fostering innovation in plant genomics and breeding.

**Endorsed by ELIXIR Nodes**: DE, BE, PT, SI, UK.

**Contact**: elixir-epan@elixir-europe.org

## Data Availability

No data is associated with this article.
