# White paper: standards for handling and analyzing plant pan-genomes

**DOI:** 10.12688/f1000research.166538.3

**Published:** 2026-05-07

**Authors:** Marc C. Heuermann, Pedro Barros, Sebastian Beier, Heidrun Gundlach, Jorge Alvarez-Jarreta, Keywan Hassani-Pak, Patrick König, Anne Fiebig, Tim Godec, Kristina Gruden, Nadja Nolte, Marko Petek, Uwe Scholz, Maja Zagorščak, Klaas Vandepoele, Michiel Van Bel

**Affiliations:** 1 Leibniz Institute of Plant Genetics and Crop Plant Research (IPK), Seeland, Saxony-Anhalt, 06466, Germany; 2 Universidade Nova de Lisboa Instituto de Tecnologia Quimica e Biologica, Oeiras, Lisbon, Portugal; 3 Forschungszentrum Jülich GmbH Institute of Bio- and Geosciences, Jülich, North Rhine-Westphalia, 52425, Germany; 4 Helmholtz Zentrum München, Neuherberg, 85764, Germany; 5 European Molecular Biology Laboratory, European Bioinformatics Institute, Wellcome Genome Campus, Hinxton, Cambridge, CB10 1SD, UK; 6 Rothamsted Research, Harpenden, England, AL52JQ, UK; 7 National Institute of Biology, Večna pot 111, Ljubljana, 1000, Slovenia; 8 Department of Plant Biotechnology and Bioinformatics, Ghent University, Technologiepark 71, Ghent, 9052, Belgium

**Keywords:** plant pan-genome, white paper, standards, quality control

## Abstract

Plant pan-genomes, which aggregate genomic sequences and annotations from multiple individuals of a species, have emerged as transformative tools for understanding genetic diversity, adaptation, and evolutionary dynamics. However, the absence of standardized practices for data generation, analysis, and sharing hinders reproducibility and interoperability. This white paper presents a harmonized framework developed by the ELIXIR E-PAN consortium, addressing nomenclature, quality control (QC), data formats, visualization, and community practices. By adopting these guidelines, researchers can enhance FAIR (Findable, Accessible, Interoperable, Reusable) compliance, foster collaboration, and accelerate translational applications in crop improvement and evolutionary biology.

## 1. Introduction

Pan-genomes capture both core genomic elements (shared across individuals) and accessory components (variable or unique to subsets), offering unprecedented resolution for studying traits such as disease resistance, environmental adaptation, and domestication ( Qin et al., 2021; Zhou et al., 2022). When sampling extends across species boundaries, the prefix super indicates a higher-level taxonomic order ( Schreiber et al., 2024). Super-pan-genomes further enable comparative analyses of clades or genera, bridging breeding applications with evolutionary insights ( Shang et al., 2022; Li et al., 2023a). Super-pan-genomes, which span multiple species, provide evolutionary context for gene family dynamics and speciation events, as demonstrated in clades like Brassicaceae ( Jiao & Schneeberger, 2020) and Solanaceae ( Alonge et al., 2022). In plant genomics, pan-genomes are vital for understanding genetic diversity, adaptation, and evolutionary dynamics, particularly given the extensive variation observed in plant species ( Schreiber et al., 2024). Despite their potential, inconsistencies in data management—such as ad hoc naming conventions, variable QC practices, and fragmented repository use—limit cross-study comparisons and data reuse.

The ELIXIR E-PAN consortium synthesizes insights from foundational studies on barley ( *Hordeum vulgare*), rice ( *Oryza sativa*), tomato ( *Solanum lycopersicum*), and Arabidopsis ( *Arabidopsis thaliana*) to propose actionable standards. These guidelines aim to unify the plant genomics community, ensuring robust, interoperable resources for breeding and evolutionary research.

## 2. Naming conventions and ontologies

### 2.1 Accession and assembly identifiers

Accession naming should adhere to MIAPPE (Minimum Information About Plant Phenotyping Experiments) standards. The Biological Material ID should incorporate institutional identifiers, followed by the accession number from germplasm catalogue or common name of the plant source/variety (e.g., IPK-Gatersleben:HOR_13170 for barley accession “Barke”) to ensure traceability (MIAPPE v1.1, Papoutsoglou et al., 2020). When complementary data regarding a specific accession is also available at external sources (e.g. Biosamples), a link to a Biological material external ID should be provided in the metadata.

Genome assembly identifiers should contain at least 4 fields—species, variety/line, project group, assembly version — separated by period (‘.’), with an optional fifth field for additional information ( Cannon et al., 2025). For example, drOrySati.Nipponbare.RicePan.1.0, which refers to the assembly of *Oryza sativa*, Nipponbare cultivar, RicePan project, version 1.0 (ToLID identifier, https://id.tol.sanger.ac.uk/, Darwin Tree of Life Consortium, 2023).

### 2.2 Gene identifiers

Gene identifiers must balance stability with biological relevance, as outlined by Cannon et al. (2025), keeping track of the annotation version, chromosome and gene ID. Their framework proposes human- and machine-readable identifiers, including the assembly names (e.g. drOrySati.Nipponbare.RicePan.1.0) with the addition of gene models like drOrySati.Nipponbare.RicePan.1.0.1.01.g000100 (assembly version 1.0, annotation version 1, chromosome 01, gene 100). To enhance this for pan-genomics, the “group” field can denote pan-genome projects (e.g., RicePan), linking multiple assemblies, while optional fields like “Hap1” or metadata tags distinguish haplotypes or accession types (e.g., wild vs. cultivated). Pangenes, representing orthologous gene clusters, can be assigned identifiers like drOrySati.RicePan.pan00001, with metadata linking to specific gene models across assemblies. Cannon et al. (2025) advocate preserving legacy identifiers via cross-references to ensure stability, avoiding disruptive renaming as new accessions are added.

### 2.3 Metadata and ontologies

A core metadata schema is critical for interoperability. Required fields to properly annotate pan-genome studies include species details such as name (TaxonID), pedigree, geographic origin, ploidy and chromosome number, as well as sequencing technology used (e.g. PacBio HiFi, Oxford Nanopore, Hi-C, Illumina), assembly pipelines (e.g., Flye ( Kolmogorov et al., 2020), hifiasm ( Cheng et al., 2024), Canu ( Koren et al., 2017), …), and assembly QC metrics (e.g., BUSCO scores ( Manni et al., 2021)). Existing ontologies such as the Sequence Ontology (SO) should be extended to include pan-genome-specific terms that describe the layouts and structures of pan-genomes ( Eilbeck et al., 2005). These can be categorized as core, shell and cloud genome genes, but these terms may depend on the number of genomes and genotypes selected ( Jayakodi et al., 2024). Any downstream comparative analysis requires open and transparent reporting on the thresholds used, so that these must be included in the metadata. Collaboration with the AgBioData Nomenclature Working Group and the Genomics Standards Consortium ( https://www.gensc.org/) ensures alignment with broader genomic standards ( Cannon et al., 2025).

### 2.4 Generalized feature identification

As pan-genome graphs grow to encompass not just core and variable genes but a full spectrum of genomic elements, we need a unified identification system. Current annotation often focuses on genes, leaving features like transposable elements, SSRs, non-coding RNAs, and regulatory motifs with inconsistent or tool-specific labels. We propose the development of a **generalized feature identifier (GFI)**. This system would provide a stable, queryable, and standardized format for any annotated feature, independent of its type or the discovery tool used. A GFI would be important for pan-genome-scale association studies and for functionally characterizing the entire “dark matter” of the genome, ensuring that a SNP in a long terminal repeat or a copy number variation in a novel ncRNA can be cataloged and compared with the same rigor as in a protein-coding gene.

## 3. Quality Control (QC) standards

### 3.1 Sequencing and assembly QC

**Figure 1.**
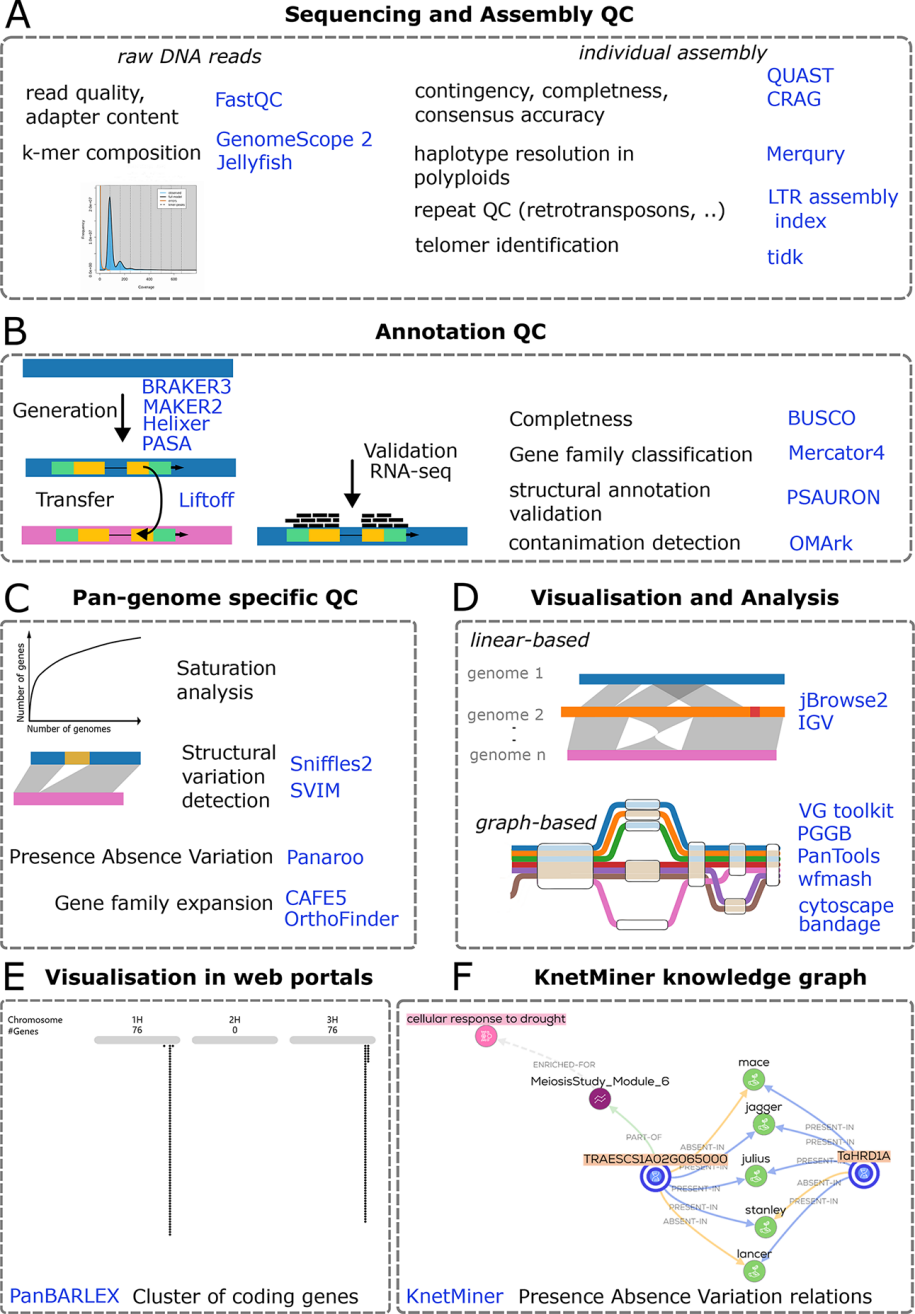
Overview of quality control, annotation, pan-genome analysis, and visualization steps across a -pan-genome project, with example tools highlighted. **A**, Sequencing and assembly QC. Raw DNA reads are screened for base quality, adapter contamination, and k-mer composition using **FastQC**, **GenomeScope 2**, and **Jellyfish**. Individual assemblies are evaluated for contiguity, completeness, and consensus accuracy with **QUAST** and **CRAG**; haplotype resolution in polyploids with **Merqury**; repeat content and assembly of long terminal repeat retrotransposons with **LTR assembly index**; and telomere identification with **tidk**. **B**, Annotation QC. Gene models are generated and refined with **BRAKER3**, **MAKER2**, **Helixer**, and **PASA**, and can be transferred between assemblies using Liftoff. Validation incorporates RNA-seq support and summary metrics including gene set completeness with **BUSCO**, gene family classification with **Mercator4**, structural annotation validation with **PSAURON**, and contamination detection with **OMArk**. **C**, Pan-genome–specific QC and discovery. Across multiple genomes, analyses include gene accumulation and saturation behavior, detection of structural variants with **Sniffles2** and **SVIM**, assessment of presence–absence variation with **Panaroo**, and tests for gene family expansion or contraction with **CAFE5** and **OrthoFinder**. **D**, Visualization and comparative analysis. Linear genome browsers support side-by-side inspection of assemblies and annotations ( **jBrowse2**, **IGV**). Graph-based frameworks represent shared and alternative haplotypes and enable mapping and variant interrogation across many genomes ( **VG toolkit**, **PGGB**, **PanTools**, **wfmash**), complemented by network and assembly graph viewers ( **cytoscape**, **bandage**). **E**, Pre-rendered web portals. Project-specific portals provide searchable tracks and summary plots for community access, exemplified by **PanBARLEX**, ( https://panbarlex.ipk-gatersleben.de/#seqcluster/BarleyCDS90_02985). **F**, Presence absence variation (PAV) relations shown in knowledge graphs produced by **KnetMiner**. Dashed boxes delineate workflow stages; icons are schematic. The listed software represents commonly used options and is not exhaustive. Abbreviations: QC, quality control; RNA-seq, RNA sequencing; PAV, presence–absence variation.

Quality control in genome assembly workflows begins with sequencing QC, where tools like FASTQC assess raw read integrity, including base quality, GC content, and adapter contamination ( Figure 1A). K-mer plots, generated via Jellyfish ( Marçais et al., 2011) paired with GenomeScope 2 ( Ranallo-Benavidez et al., 2020), provide insights into genome complexity, such as ploidy, heterozygosity, and repetitive element profiles ( Figure 1A). For individual assembly QC, QUAST ( Mikheenko et al., 2023) is recommended for evaluating contiguity metrics (e.g., N50, L50) and is particularly effective for comparing multiple assemblies of diploid genomes, while CRAQ ( Li et al., 2023) excels in assessing consensus accuracy and structural errors in polyploid genomes due to its sensitivity to haplotype-specific misassemblies ( Figure 1A). When results from QUAST and CRAQ conflict (e.g., differing contig counts due to haplotype collapsing), users should prioritize CRAQ for polyploid assemblies and cross-validate with raw read alignments (e.g., using Minimap2) to resolve discrepancies. Merqury ( Rhie et al., 2020) further validates haplotype resolution in polyploid or heterozygous genomes (e.g., wheat, potato) by comparing k-mer spectra between raw reads and assemblies, offering a robust check for completeness and phasing errors ( Figure 1A). For repeat quality control, the LTR Assembly Index (LAI; Ou et al., 2018) assesses the completeness of long terminal repeat retrotransposons, while *tidk* ( Brown et al., 2025) detects telomeric motifs to evaluate chromosomal end-to-end integrity ( Figure 1A). When results from these tools conflict, LAI generally provides a more reliable indicator of assembly quality. Even in high-quality plant genomes assembled from long reads, some chromosome ends may still lack detectable telomeric repeats. Wang and Wang (2023) propose a framework with eight metrics for quality control in genome assembly, covering the three core dimensions: contiguity (N50 + contig/chromosome ratio), completeness (overall k-mer-based, BUSCO gene space, tandem repeats and organelle genomes) and correctness (error rates at base and structural level)–along with recommended values for a finished assembly.

### 3.2 Annotation QC

Annotation pipelines must be documented alongside assembly strategies. These may include gene model integration pipelines like MAKER2 ( Holt & Yandell, 2011), PASA ( Haas et al., 2003) or BRAKER3 ( Gabriel et al., 2024), while Helixer ( Stiehler et al., 2020) is recommended for ab initio prediction in non-model organisms due to its deep learning-based approach ( Figure 1B). Liftoff ( Shumate & Salzberg, 2021) is ideal for annotation transfer between closely related species and should be part of a standard annotation pipeline ( Figure 1B). Use versioned workflows (e.g., Snakemake ( Köster & Rahmann, 2012), or Nextflow ( Di Tommaso et al., 2017)) to ensure reproducibility, provenance tracking, and portability. Transcriptomic data (RNA-Seq) from multiple tissues (e.g., roots, shoots) and stress conditions (e.g., drought, disease) with sufficient read coverage validates gene models, especially for accessory genes lacking orthologs ( Qin et al., 2021). Long-read RNA sequencing technologies are recommended to recover full-length transcripts and accurately characterize alternative isoforms. For structural annotation QC, BUSCO ( Manni et al., 2021) assesses gene space completeness using lineage-specific datasets that can be adjusted for polyploid genomes ( Figure 1B). Mercator4 ( Bolger et al., 2021) assigns functional categories based on the MapMan bin system and is useful for identifying missing functions in a single genome ( Figure 1B). PSAURON ( Sommer et al., 2025) validates structural annotations, and OMArk ( Nevers et al., 2025) detects contamination via evolutionary consistency checks ( Figure 1B). In cases where evaluation tools disagree (e.g., BUSCO reports missing genes but PSAURON suggests completeness), integrating RNA-Seq support and orthology evidence provides a more reliable basis for resolving such discrepancies ( Veeckman et al., 2016).

### 3.3 Pan-genome-specific QC

Pan-genome completeness requires saturation analysis, where gene accumulation curves assess whether additional accessions contribute novel genes ( Tettelin et al., 2005). For species with varying ploidy levels (e.g., diploid vs. polyploid barley), a minimum of 10–20 accessions is typically required for diploid species to approach saturation, while polyploid species may need 30–50 accessions due to increased gene content complexity ( Jayakodi et al., 2024). Users should plot accumulation curves using tools like Panaroo and evaluate saturation by fitting models (e.g., Heap’s Law) to confirm diminishing returns in gene discovery ( Figure 1C). For species like barley, benchmark datasets of 100+ conserved genes enable orthology tool validation ( Jayakodi et al., 2024). OrthoFinder ( Emms & Kelly, 2019) and CAFE5 ( Mendes et al., 2020) facilitate gene family expansion and contraction analyses, providing insights into evolutionary dynamics ( Figure 1C). Structural variant detection, using Sniffles2 ( Smolka et al., 2024) for long-read data or SVIM ( Heller & Vingron, 2019) for short-read data, quantifies indels and inversions ( Qin et al., 2021) ( Figure 1C). When tools like Sniffles2 and SVIM yield conflicting variant calls, users should integrate multi-platform data (e.g., combining long- and short-read alignments) and prioritize calls supported by higher read depth or mapping quality. Presence-absence variation (PAV) detection via Panaroo or PAV-specific pipelines is critical for identifying variable gene content tied to phenotypic diversity ( Tonkin-Hill et al., 2020).

### 3.4 Inter-species Super-pan-genome QC

Although the toolkits are overlapping, there are notable differences in quality control (QC) practices between intra-species pan-genome analyses and inter-species super-pan-genome analyses. These differences stem from the scope: closely related germplasm within a narrow framework versus divergent species across different gene pools. Super-pan-genome QC demands stronger per-assembly validation (deeper anchoring, transcript mapping, Hi-C on selected genomes) to accommodate inter-species variability in repeats, genome size, and structure, preventing error propagation in cross-species analyses like orthogroup clustering or collinearity. Both rely on annotation QC for gene-space completeness and read-mapping rates, but super-pan-genomes often add more comparative metrics (e.g., core vs. dispensable gene proportions, SV hotspot validation) and handle greater technical challenges from divergent repeats or centromeres.

## 4. Data formats and sharing

### 4.1 File formats

• **Raw data**: Assemblies must be submitted in FASTA format with headers containing unique sequence identifiers (e.g., >chr01, >chr02). Annotations must be provided in GFF3 or GTF format (compliant with Sequence Ontology), with the sequence IDs in the first column exactly matching the sequence identifiers used in the FASTA headers.
• **Derived data**: Structural variants in VCF/BCF, orthogroups in TSV (cluster ID + member gene), and graph-based representations (GFA format) for complex pan-genomes ( Li et al., 2020).

### 4.2 Repositories

Centralized repositories would archive versioned datasets (e.g., Barley v2, Rice v1.5) with DOI-based identifiers (DataCite). Public deposition in INSDC (raw reads and assembly, https://www.insdc.org/), Ensembl (annotations, see documentation of Ensembl, 2025, https://beta.ensembl.org/), and the National Genomics Data Center, which is a part of the China National Center for Bioinformation (CNCB) (CNCB-NGDC, https://ngdc.cncb.ac.cn/), ensures global accessibility (ENA Documentation, 2025).

### 4.3 Metadata requirements

Mandatory metadata fields include sequencing technology and coverage (e.g., PacBio HiFi, Oxford Nanopore), assembly method (e.g., Flye, Hifiasm), accession provenance (BioSample IDs), and software versioning of all software and pipelines used. Missing metadata must be addressed via enforced submission guidelines.

For pangenome datasets, additional metadata fields are critical to ensure traceability and interoperability across studies. These should include the species name and NCBI Taxonomy ID, pangenome version and build date, and a complete list of constituent genomes with corresponding assembly accessions, strain names, and versions. Furthermore, metadata should describe the methods and parameters used to construct the pangenome.

Capturing this information in structured formats such as JSON-LD or RO-Crate ( Peroni et al., 2022) would align pangenome submissions with broader FAIR data principles and facilitate integration with knowledge graphs and comparative genomics resources.

### 4.4 Practical implementation challenges and best practices for community adoption

The proposed standards strongly support FAIR compliance, but their community-wide adoption faces practical challenges that require explicit guidance. Maintaining consistent metadata across independent projects can remain an obstacle. Even small differences in accession naming, software versioning, or provenance tracking can render datasets incompatible when merged, preventing reliable cross-study comparisons of presence-absence variation (PAV) or orthology. Another challenge is the limited native support for advanced formats in major repositories. INSDC and Ensembl readily accept FASTA, GFF3, and VCF but offer only partial or no built-in support for pan-genome graphs (GFA) or structured metadata (JSON-LD/RO-Crate).

To address this, it would be recommended to submit a minimal compliant package (FASTA + GFF3 + VCF + core metadata) to INSDC/Ensembl and attaching a single RO-Crate archive containing the full GFA, detailed provenance (Snakemake or Nextflow workflow files), and validation reports. For legacy datasets that pre-date these guidelines, a pragmatic workflow is advised: reconstruct missing fields from BioSample records, re-annotate genes with Liftoff against the current reference, and wrap the updated resource in an RO-Crate “patch” that links back to the original accession. These standards are equally essential for inter-species super-pan-genomes, where consistent nomenclature, metadata, and graph formats across species boundaries are critical to enable reliable comparative and evolutionary analyses of clades or genera.

By implementing these targeted mitigations—community metadata templates, RO-Crate wrappers, and hybrid deposition— adoption barriers can be overcome while preserving the value of both new and legacy pan-genomes.

## 5. Visualization and analysis guidelines

### 5.1 Visualization tools

Plant pan-genomes capture a species’ full genomic diversity, constructed using either linear-based or graph-based methods, each with distinct strengths and limitations. To provide a clearer comparison, linear-based approaches are divided into two distinct categories: sequence-based and gene-based analyses.

**Sequence-based linear analysis** involves aligning multiple genomes to a single reference or consensus sequence to identify sequence-level variations, such as single-nucleotide polymorphisms (SNPs) and insertions/deletions (indels). This process typically employs variant callers like GATK ( McKenna et al., 2010) or freebayes ( Garrison & Marth, 2012) to detect SNPs and indels from whole-genome alignments. These methods are computationally efficient and compatible with visualization tools like JBrowse2 ( Diesh et al., 2023) or IGV ( Robinson et al., 2023) for synteny and variant visualization ( Figure 1D). Web-portals such as PanBARLEX ( PanBARLEX - Barley Pangenome Explorer) enable pan-genome research by providing searchable and pre-rendered visualizations ( Figure 1E). However, reference bias in sequence-based linear approaches can limit their ability to capture complex structural variations, particularly in repetitive or polyploid plant genomes.

**Gene-based linear analysis** focuses on inferring orthology and identifying gene-level presence/absence variations (PAVs) using tools like OrthoFinder ( Emms & Kelly, 2019) or Ensembl Compara ( Dyer et al., 2025). These tools analyze annotated gene sets to determine the pan-gene repertoire, identifying core and accessory genes across a species. While effective for gene-level PAV detection, these methods do not directly address sequence-level variations like SNPs or indels, requiring separate workflows for comprehensive analysis. Orthology inference tools must be benchmarked using inflation value sweeps to minimize false positives ( Emms & Kelly, 2019). Visualization of gene-level PAVs can be achieved through UpSet plots or as presence/absence relationships in KnetMiner knowledge graphs ( Hassani-Pak et al., 2021) ( Figure 1F).

In contrast, **graph-based approaches** model genomes as interconnected nodes (shared regions) and edges (SNPs, indels, and structural variants) using tools like VG Toolkit ( Hickey et al., 2020), PGGB ( Garrison et al., 2024), PanTools ( Jonkheer et al., 2022), or wfmash ( Guarracino et al., 2021) ( Figure 1D). These methods integrate both sequence-level and structural variations in a single framework, offering an unbiased, comprehensive view of genomic diversity. They are particularly suited for complex genomes, such as tomato ( Zhou et al., 2022). Visualization tools like Bandage ( Wick et al., 2015) or Cytoscape ( Shannon et al., 2003) are used to represent structural complexity, though these approaches are computationally intensive and require specialized expertise ( Figure 1D).

In summary, sequence-based linear methods excel in rapid SNP and indel detection but are limited by reference bias, while gene-based linear methods are ideal for pan-gene analysis but require separate homology-based workflows. Graph-based approaches unify both gene-level and structural variation analyses, offering greater flexibility for complex genomes despite higher computational demands. As computational resources and tools advance, graph-based methods are becoming more accessible, enhancing plant pan-genome studies as demonstrated in rice ( Qin et al., 2021).

### 5.2 Integrative analysis best practices

The integration of pangenomic information into crop improvement remains challenging, despite its potential to illuminate the genetic basis of agronomic traits. Pangenomes reveal extensive structural polymorphisms and gene content diversity across accessions, yet these findings often remain siloed from other key data sources such as GWAS and QTL mappings, gene expression profiles, gene regulation, functional annotations, and published literature. Without coherent integration, researchers face difficulties in linking genomic variation to phenotype and in distinguishing biologically meaningful signals from background noise. Bridging these data types requires frameworks capable of harmonizing heterogeneous evidence, tracking provenance, and enabling transparent reasoning across molecular, phenotypic, and bibliographic domains.

Platforms such as KnetMiner ( Hassani-Pak et al., 2021, https://knetminer.com) address these challenges by synthesizing pangenomic, association, omics, and literature-derived evidence within a unified knowledge graph. This integrative approach allows relationships among genes, traits, and pathways to be explored in context, supporting AI-assisted hypothesis generation and candidate gene prioritization. By providing explainable connections between diverse evidence sources, KnetMiner exemplifies how knowledge graph technologies can transform FAIR yet fragmented genomic data into a coherent foundation for evidence-based crop breeding.

## 6. Case studies

**Barley Pan-genome** ( Jayakodi et al., 2024): The IPK barley pan-genome v2, encompassing 76 accessions, faced significant challenges in diploid genome assembly due to the crop’s complex genetic structure. The adoption of automated quality control (QC) pipelines, alongside validation gene sets, was critical to ensuring reproducibility and accuracy. By streamlining QC processes, the project achieved robust assembly outcomes, enabling reliable downstream analyses for barley breeding programs. Without such standards, the project risked fragmented datasets, highlighting the necessity of automation for handling complexity. The barley pan-genome reveals how structural variants and copy number variations at complex loci can be harnessed for plant breeding by enabling the discovery and targeted deployment of novel alleles for disease resistance (Mla), malting quality (amy1_1), plant architecture (HvTB1), and trichome development (HvSRH1). It enhances GWAS and genetic mapping by improving short-read alignment rates, capturing a wider range of haplotypes, and resolving presence/absence variants that linear reference genomes miss. The pan-genome provides insights into crop evolution by showing post-domestication gains in allelic diversity, supporting better understanding of adaptation to agricultural environments.

**Rice Pan-genome (** Qin et al., 2021): Analysis of 31 rice accessions using Sniffles revealed hidden structural variations critical for understanding genetic diversity. However, the absence of standardized QC metrics initially led to discrepancies in variant calling, complicating comparisons across accessions. The project’s success in identifying novel variations was enhanced by post-hoc implementation of rigorous QC protocols, which improved variant validation and reproducibility. The rice pan-genome enhances genome-wide association studies (GWAS) by enabling the detection of phenotype-associated SVs, including a 987-bp LTR insertion linked to early leaf senescence, that remain undetectable using only SNPs or a single linear reference genome. This case underscores the need for predefined, community-wide QC standards to ensure consistency in pan-genome analyses, as their absence delayed insights into rice diversity and potential breeding applications.

**Tomato Pan-genome** ( Zhou et al., 2022): The tomato pan-genome, comprising 838 genomes, utilized a graph-based representation to resolve complex structural variants, directly informing breeding strategies for disease resistance. It improves GWAS power through better variant detection and resolution of incomplete LD, allelic, and locus heterogeneity, while capturing 24% more missing heritability (0.41 vs 0.33) for thousands of expression and metabolite traits. Plant breeding benefits from the identification of causal structural variations (SVs) for key traits such as soluble solids content, enabling more precise marker-assisted selection and genomic selection (using a specially developed, cost-effective SV capture array), and supports genome editing through comprehensive gene and sgRNA resources. The adoption of standardized graph-based assembly tools ensured accurate representation of genetic diversity, overcoming limitations of linear reference genomes. This standardized approach facilitated the identification of novel resistance genes, significantly advancing breeding outcomes. Without such standards, the project could have faced misassembled variants, reducing its utility for applied breeding. This case exemplifies how standardized frameworks enhance the resolution of complex genomic data for practical applications.

**Arabidopsis (** Jiao & Schneeberger 2020; Zhong et al., 2025): Annotation gene naming and transfer across Arabidopsis MAGIC founders using Liftoff achieved cross-accession consistency. The use of standardized annotation pipelines ensured accurate gene mapping, enabling robust multi-omic and pan-genomic comparisons. Pan-genome analysis of chromosome-level assemblies reveals 5.1–6.5 Mb of accession-specific sequences, approximately 1,900 non-reference genes, and copy-number variations affecting around 5,000 genes, showing that a single reference genome captures only the core genome of about 105 Mb with roughly 24,000 genes, while the full species pan-genome reaches approximately 135 Mb with about 30,000 genes. Although Arabidopsis itself is not a direct breeding target, this resource serves as a translational model for crop improvement by identifying rearrangement hotspots enriched for biotic stress-response genes and R-genes that can accelerate breeding for pathogen resistance and stress tolerance in crops such as rice, wheat, and tomato. This standardization was pivotal in identifying functional genomic variations within the population, supporting downstream genetic studies. In contrast, earlier Arabidopsis pan-genome efforts lacking such standardized tools faced annotation inconsistencies, which hindered comparative analyses. This case highlights how standardized naming conventions and annotation transfer tools like Liftoff are essential for ensuring reliable and reproducible pan-genomic insights.

In all four case studies, intra-species pan-genomes are analyzed. These differ from inter-species super-pan-genomes in terms of their potential utility and their contribution to downstream analyses. Pan-genome analyses excel at capturing fine-grained genetic variation within germplasm and its immediate wild progenitor. This directly supports downstream breeding applications like high-resolution genomic selection, marker-assisted selection using SV markers, and precise genome editing within elite breeding material.

In contrast, inter-species super-pan-genomes, exemplified by a super-pan-genome in tomato ( Li et al. 2023a), integrate chromosome-scale assemblies across multiple wild and cultivated tomato species in the *Solanum* section *Lycopersicon*. They reveal a much broader gene repertoire (only ~54% core genes) and tens of thousands of wild-specific SVs and presence/absence variations that are largely absent in narrow intra-species analyses due to domestication bottlenecks. Li et al. (2023a) was able to increase tomato fruit yield by overexpressing a wild tomato gene that had been identified as part of the super-pan-genome analysis.

Together, intra-species approaches optimize precision and power for current cultivated populations, while super-pan-genomes provide a richer reservoir of untapped diversity. The two strategies are complementary: the former refines within-crop improvement, and the latter expands the genetic base for long-term resilience and innovation in crop breeding; the potential of super-pan-genomes in plant breeding is tremendous ( Raza et al., 2026). The ongoing challenges in pan-genome analyses, computational demands, and the need to develop faster, and more efficient tools are discussed in greater detail in a recent review article ( Jayakodi et al., 2025).

## 7. Future directions

• **Artificial Intelligence**: Tools like DeepVariant ( Poplin et al., 2018) will enhance variant calling in polyploid genomes. Detection of other genomic features, such as repeat elements, regulatory elements, and binding sites, will be enabled and refined using foundational models, as demonstrated in recent high-impact studies. For instance, BigRNA predicts tissue-specific RNA expression and identifies regulatory elements like microRNA and protein binding sites with high accuracy ( Celaj et al., 2023). Similarly, Evo 2 detects transcription factor binding sites and exon-intron boundaries across diverse genomes ( Brixi et al., 2025), while models like DNABERT ( Ji et al., 2021) and Enformer ( Avsec et al., 2021) excel in promoter prediction and variant effect analysis ( Li et al., 2024). These advancements highlight the transformative potential of foundational models in refining genomic feature detection, particularly for complex polyploid genomes.
• **Cross-species standards**: Develop clade-wide frameworks (e.g., Brassicaceae) to unify super-pan-genome analyses.
• **Community engagement**: ELIXIR hackathons will refine workflows and ontology terms, ensuring adaptability to technological advances.

## 8. Conclusion

This white paper establishes a community-driven framework for plant pan-genome research. By adopting these guidelines, researchers can ensure data interoperability, reproducibility, and translational impact. The E-PAN consortium calls for global collaboration to iteratively refine these standards, fostering innovation in plant genomics and breeding.

**Endorsed by ELIXIR Nodes**: DE, BE, PT, SI, UK.

## Data Availability

No data is associated with this article.
