# Supplementary material for: White paper: standards for handling and analyzing plant pan-genomes
Source: F1000Res. 2026 May 7;14:739. Originally published 2025 Jul 28. [Version 3] doi: 10.12688/f1000research.166538.3 (PMC12739369; doi:10.12688/f1000research.166538.3)
Supplement: Peer review history

# Reviewer report: Reviewer response for version 3

**DOI:** 10.5256/f1000research.200310.r482397

**Published:** 2026-05-08

**Authors:** Jianping Xu

**Affiliations:** 1 Department of Biology, McMaster University, Hamilton, Canada

I'm very happy to endorse the newly revised version for indexing.

Is the review written in accessible language?

Yes

Are all factual statements correct and adequately supported by citations?

Yes

Are the conclusions drawn appropriate in the context of the current research literature?

Yes

Is the topic of the review discussed comprehensively in the context of the current literature?

Yes

Reviewer Expertise:

population genetics and genomics, with a focus on fungi

I confirm that I have read this submission and believe that I have an appropriate level of expertise to confirm that it is of an acceptable scientific standard.

# Reviewer report: Reviewer response for version 2

**DOI:** 10.5256/f1000research.191128.r436510

**Published:** 2025-12-30

**Authors:** Jianping Xu

**Affiliations:** 1 Department of Biology, McMaster University, Hamilton, Canada

This is an excellent set of recommendations for handling and analyzing plant pan-genomes. I have only three minor comments/questions for authors' considerations.

 1. Should there be a clearly stated minimum standard for a genome to be included in plant pan-genome analyses? In the examples/case studies provided, what were the inclusion criteria and is there an emerging consensus?

 2. Even though the abstract included "Super-pan-genomes", aside from the tomato case study example at the very end, very little attention was paid to this topic in the main text. Should there be additional criteria for super-pan-genome studies on top of what's recommended for pan-genome analyses?

 3. This White Paper listed INSDC and Ensembl as the suggested data repositories. Given the increasing importance of genomic and pan-genomic data from China and the depositions of such data in the Chinese National Genomic Data Center (https://ngdc.cncb.ac.cn/), I think it's important to include that database as a suggested repository for pan-genome datasets.

 A minor editorial comment: two citations were included in the abstract of this paper. Can the abstract in this journal include citations?

Is the review written in accessible language?

Yes

Are all factual statements correct and adequately supported by citations?

Yes

Are the conclusions drawn appropriate in the context of the current research literature?

Yes

Is the topic of the review discussed comprehensively in the context of the current literature?

Yes

Reviewer Expertise:

population genetics and genomics, with a focus on fungi

I confirm that I have read this submission and believe that I have an appropriate level of expertise to confirm that it is of an acceptable scientific standard, however I have significant reservations, as outlined above.

# Response

**Published:** 2026-04-24

**Authors:** Marc Christian Heuermann

1. Should there be a clearly stated minimum standard for a genome to be included in plant pan-genome analyses? In the examples/case studies provided, what were the inclusion criteria and is there an emerging consensus?

 Answer: Thank you for your suggestions. We added a sentence in Chapter 3.1, referred to a publication by Wang & Wang 2023, and provided a summary of their proposed framework for quality control in genome assembly, which also contains threshold recommendations.

 2. Even though the abstract included "Super-pan-genomes", aside from the tomato case study example at the very end, very little attention was paid to this topic in the main text. Should there be additional criteria for super-pan-genome studies on top of what's recommended for pan-genome analyses?

 Answer: In the “Case studies” section, we have discussed in greater detail the difference between intra-species pan-genomes and inter-species super-pan-genomes, as well as their complementary nature. In addition, we have included a new Section 3.4 to explain in more detail the differences in quality control when analyzing an inter-species super-pan-genome.

 3. This White Paper listed INSDC and Ensembl as the suggested data repositories. Given the increasing importance of genomic and pan-genomic data from China and the depositions of such data in the Chinese National Genomic Data Center (https://ngdc.cncb.ac.cn/), I think it's important to include that database as a suggested repository for pan-genome datasets.

 Answer: Thank you very much for pointing that out. This would indeed improve the overall visibility and reliability of data storage, which is why we have included this in the manuscript.

 A minor editorial comment: two citations were included in the abstract of this paper. Can the abstract in this journal include citations?

 Answer: Indeed, according to the guidelines, this is not permitted. We moved the whole sentence with citation into the introduction section.

# Reviewer report: Reviewer response for version 2

**DOI:** 10.5256/f1000research.191128.r434365

**Published:** 2025-12-30

**Authors:** Rutwik Barmukh

**Affiliations:** 1 Centre for Crop and Food Innovation, Murdoch University, Murdoch, Western Australia, Australia

This white paper makes an impressive effort to harmonize methodological practices for plant pan-genome construction, analysis, and sharing, as pan-genomic resources are rapidly increasing across different crop species. The manuscript is highly relevant and addresses a clear need within the plant genomics community. However, to function effectively as a widely adopted standards document, the manuscript would benefit from stronger justification of recommended practices and better consideration of downstream use cases and implementation challenges. The manuscript’s clarity, usability, and long-term impact will significantly improve after addressing some issues highlighted below.

 1. Although the manuscript highlights the importance of the proposed standards for improving FAIR compliance, the practical aspects of implementing these standards in real-world scenarios can be explored further. For instance, challenges related to community-wide adoption, such as maintaining consistent metadata across projects and the limited support for certain recommended formats (e.g. GFA, JSON-LD) in existing repositories, deserve more explicit discussion. In addition, suggestions for handling legacy datasets that lack complete or standardized metadata would be beneficial. Providing concrete examples of common pitfalls (e.g., interoperability failures caused by inconsistent metadata) along with potential mitigation approaches would further strengthen the paper.

 2. While the manuscript precisely captures genomic and computational standards, it currently lacks guidance on how pan-genome outputs should interface with downstream applications that are most relevant to plant breeders and geneticists (e.g., GWAS, QTL mapping, selection decisions, etc.). A subsection linking pan-genome data to common downstream analyses can be added. Also, example schemas or pipelines showing how standardized pan-genome representations improve trait mapping or selection decisions in practice can be included.

 3. The Case Studies section highlights several key projects, but it reads as descriptive rather than analytical. It would be more convincing if this section contained quantitative comparisons showing how applying the proposed standards increased reproducibility, efficiency, or interpretability over previous, unstandardized approaches.

Is the review written in accessible language?

Partly

Are all factual statements correct and adequately supported by citations?

Yes

Are the conclusions drawn appropriate in the context of the current research literature?

Yes

Is the topic of the review discussed comprehensively in the context of the current literature?

Yes

Reviewer Expertise:

Crop genomics, bioinformatics, molecular breeding

I confirm that I have read this submission and believe that I have an appropriate level of expertise to confirm that it is of an acceptable scientific standard, however I have significant reservations, as outlined above.

# Response

**Published:** 2026-04-24

**Authors:** Marc Christian Heuermann

1. Although the manuscript highlights the importance of the proposed standards for improving FAIR compliance, the practical aspects of implementing these standards in real-world scenarios can be explored further. For instance, challenges related to community-wide adoption, such as maintaining consistent metadata across projects and the limited support for certain recommended formats (e.g. GFA, JSON-LD) in existing repositories, deserve more explicit discussion. In addition, suggestions for handling legacy datasets that lack complete or standardized metadata would be beneficial. Providing concrete examples of common pitfalls (e.g., interoperability failures caused by inconsistent metadata) along with potential mitigation approaches would further strengthen the paper.

 Answer: Thank you for your suggestions. We have added a new subsection 4.4 (“Practical implementation challenges and best practices for community adoption”) to Section 4. This subsection explicitly discusses challenges such as maintaining consistent metadata across independent projects, the current limited native support for GFA and JSON-LD/RO-Crate formats in major repositories (INSDC and Ensembl), and the difficulties posed by legacy datasets that pre-date these standards.

 2. While the manuscript precisely captures genomic and computational standards, it currently lacks guidance on how pan-genome outputs should interface with downstream applications that are most relevant to plant breeders and geneticists (e.g., GWAS, QTL mapping, selection decisions, etc.). A subsection linking pan-genome data to common downstream analyses can be added. Also, example schemas or pipelines showing how standardized pan-genome representations improve trait mapping or selection decisions in practice can be included.

 Answer: We have added discussions and background information to the “Case studies” section to use them as examples of how pan-genome analyses can contribute to downstream analyses.

 3. The Case Studies section highlights several key projects, but it reads as descriptive rather than analytical. It would be more convincing if this section contained quantitative comparisons showing how applying the proposed standards increased reproducibility, efficiency, or interpretability over previous, unstandardized approaches.

 Answer: We have expanded the “Case studies” section to provide more background information and explanations regarding how pan-genome analysis has significantly improved and contributed to the downstream analyses. However, providing quantitative comparisons is, in our opinion, out of scope for this review paper.

# Reviewer report: Reviewer response for version 2

**DOI:** 10.5256/f1000research.191128.r433712

**Published:** 2025-12-24

**Authors:** Xiaoming Xie

**Affiliations:** 1 Wheat Genetics and Genomics Center, China Agricultural University College of Agronomy and Biotechnology (Ringgold ID: 200630), Beijing, Beijing, China

I have no further comments to make.

Is the review written in accessible language?

Partly

Are all factual statements correct and adequately supported by citations?

Yes

Are the conclusions drawn appropriate in the context of the current research literature?

Yes

Is the topic of the review discussed comprehensively in the context of the current literature?

Yes

Reviewer Expertise:

wheat pangenome, gene-based pangenome, comparative genomics

I confirm that I have read this submission and believe that I have an appropriate level of expertise to confirm that it is of an acceptable scientific standard.

# Reviewer report: Reviewer response for version 2

**DOI:** 10.5256/f1000research.191128.r433713

**Published:** 2025-11-25

**Authors:** Sunil Kumar Sahu

**Affiliations:** 1 State Key Laboratory of Genome and Multi-omics Technologies, BGI Research, Shenzhen, China

I am happy with the author's thorough revision and detailed reponse. I have no further comments

Is the review written in accessible language?

Yes

Are all factual statements correct and adequately supported by citations?

Yes

Are the conclusions drawn appropriate in the context of the current research literature?

Yes

Is the topic of the review discussed comprehensively in the context of the current literature?

Yes

Reviewer Expertise:

Plant genomics and evolution

I confirm that I have read this submission and believe that I have an appropriate level of expertise to confirm that it is of an acceptable scientific standard.

# Reviewer report: Reviewer response for version 1

**DOI:** 10.5256/f1000research.183537.r404154

**Published:** 2025-09-04

**Authors:** Xiaoming Xie

**Affiliations:** 1 Wheat Genetics and Genomics Center, China Agricultural University College of Agronomy and Biotechnology (Ringgold ID: 200630), Beijing, Beijing, China

This white paper by Heuermann *et al.* presents a timely and comprehensive framework aiming to establish community-wide standards for plant pan-genome analysis. The authors cover critical aspects from nomenclature and quality control to data sharing and visualization. This work represents a valuable and necessary initiative to promote FAIR principles in a rapidly evolving field. However, several major revisions are required to enhance its practical utility, scientific rigor, and overall impact before it can be endorsed as a foundational guide for the community.

 Major comments

 1. Lack of Practical Guidance and Actionable Workflows. While the paper provides an exhaustive list of tools and standards, it currently functions more as a catalogue than a practical guide. A researcher new to the field would struggle to navigate the options and select the most appropriate methodology for their specific project. To address this, the authors should incorporate one or more decision-tree figures or summary tables that guide users based on their specific research context (e.g., species ploidy, data type, biological question). Such a resource would transform this document from a simple list into an indispensable, actionable guide.

 2. Understated Importance of the Generalized Feature Identifier (GFI) Concept. The proposal of a 'Generalized Feature Identifier' (GFI) in the 'Future Directions' section is a highly innovative and critical idea. It elegantly addresses a major bottleneck in functionally annotating the non-genic 'dark matter' of pan-genomes, which is often overlooked. However, its placement as a future thought diminishes its significance. This concept should be introduced much earlier in the manuscript, possibly in the nomenclature section, and framed as a core recommendation of this white paper to highlight its forward-thinking contribution.

 3. Imprecise Comparison of Linear- vs. Graph-Based Approaches. The distinction between linear- and graph-based pan-genomes in Section 5.1 is crucial, but the current description of linear approaches could be refined for greater accuracy and clarity. The manuscript conflates two distinct types of 'linear-based' analyses. It states that these approaches identify sequence-level variations (SNPs, indels) as well as gene-level presence/absence variations (PAVs). However, the tools cited as examples, such as OrthoFinder and Ensembl Compara, are primarily used for inferring orthology and identifying gene-level PAVs. They are not the primary tools for calling SNPs and small indels from whole-genome alignments (which typically involves a separate workflow with variant callers like GATK against a linear reference). This conflation creates an imprecise comparison with graph-based approaches, which are fundamentally designed to model sequence-level variation directly. To improve this section, we recommend the authors explicitly distinguish between: (a) Sequence-based linear analysis: Aligning multiple genomes to a single linear reference to call SNPs and indels. (b) Gene-based linear analysis: Using orthology inference tools on annotated gene sets to determine the pan-gene repertoire and gene-level PAVs. By separating these two concepts, the manuscript can provide a more accurate and nuanced comparison, highlighting how graph-based pan-genomes aim to integrate both types of variation in a way that requires distinct workflows in a traditional linear framework.

 Minor comments

 1. The authors should adopt a more authoritative tone appropriate for a standards paper. Phrases like "probably more suited" (Section 2.3) should be replaced with definitive recommendations (e.g., "we recommend the use of...") to provide clear guidance.

 2. In Section 4.3 (Metadata requirements), the list of mandatory fields should be expanded to include the specific versions of all software and pipelines used. This is essential for ensuring full reproducibility of the analyses.

 3. The case studies in Section 6 are too brief to be impactful. Each case should be slightly expanded to illustrate how the application (or lack thereof) of the proposed standards directly impacted the project's outcomes, challenges, or successes. This would provide concrete evidence for the importance of the proposed standards.

 4. Regarding the classification of genes into "core, shell, and cloud" (Section 2.3), it is crucial to state that the specific percentage thresholds used for these definitions must be explicitly reported in the metadata, as they can significantly impact downstream comparative analyses.

Is the review written in accessible language?

Partly

Are all factual statements correct and adequately supported by citations?

Yes

Are the conclusions drawn appropriate in the context of the current research literature?

Yes

Is the topic of the review discussed comprehensively in the context of the current literature?

Yes

Reviewer Expertise:

wheat pangenome, gene-based pangenome, comparative genomics

I confirm that I have read this submission and believe that I have an appropriate level of expertise to confirm that it is of an acceptable scientific standard, however I have significant reservations, as outlined above.

# Response

**Published:** 2025-11-10

**Authors:** Marc Christian Heuermann

**Reviewer 2**

 Xiaoming Xie ( https://orcid.org/0000-0002-7925-4964), Wheat Genetics and Genomics Center, China Agricultural University College of Agronomy and Biotechnology (Ringgold ID: 200630), Beijing, Beijing, China

 Approved with Reservations

 This white paper by Heuermann *et al.* presents a timely and comprehensive framework aiming to establish community-wide standards for plant pan-genome analysis. The authors cover critical aspects from nomenclature and quality control to data sharing and visualization. This work represents a valuable and necessary initiative to promote FAIR principles in a rapidly evolving field. However, several major revisions are required to enhance its practical utility, scientific rigor, and overall impact before it can be endorsed as a foundational guide for the community.

  Major comments

  1. Lack of Practical Guidance and Actionable Workflows. While the paper provides an exhaustive list of tools and standards, it currently functions more as a catalogue than a practical guide. A researcher new to the field would struggle to navigate the options and select the most appropriate methodology for their specific project. To address this, the authors should incorporate one or more decision-tree figures or summary tables that guide users based on their specific research context (e.g., species ploidy, data type, biological question). Such a resource would transform this document from a simple list into an indispensable, actionable guide.

**Answer: Thank you for your insightful comments. Both reviewers raised this point, prompting us to create Figure 1, which clearly illustrates how each tool fits into the pan-genome analysis workflow. We have now cross-referenced the figure in both Section 3 and Section 5.**

  2. Understated Importance of the Generalized Feature Identifier (GFI) Concept. The proposal of a 'Generalized Feature Identifier' (GFI) in the 'Future Directions' section is a highly innovative and critical idea. It elegantly addresses a major bottleneck in functionally annotating the non-genic 'dark matter' of pan-genomes, which is often overlooked. However, its placement as a future thought diminishes its significance. This concept should be introduced much earlier in the manuscript, possibly in the nomenclature section, and framed as a core recommendation of this white paper to highlight its forward-thinking contribution.

**Answer: We do agree with the assessment and positioned the GFI concept now as paragraph 2.4.**

  3. Imprecise Comparison of Linear- vs. Graph-Based Approaches. The distinction between linear- and graph-based pan-genomes in Section 5.1 is crucial, but the current description of linear approaches could be refined for greater accuracy and clarity. The manuscript conflates two distinct types of 'linear-based' analyses. It states that these approaches identify sequence-level variations (SNPs, indels) as well as gene-level presence/absence variations (PAVs). However, the tools cited as examples, such as OrthoFinder and Ensembl Compara, are primarily used for inferring orthology and identifying gene-level PAVs. They are not the primary tools for calling SNPs and small indels from whole-genome alignments (which typically involves a separate workflow with variant callers like GATK against a linear reference). This conflation creates an imprecise comparison with graph-based approaches, which are fundamentally designed to model sequence-level variation directly. To improve this section, we recommend the authors explicitly distinguish between: (a) Sequence-based linear analysis: Aligning multiple genomes to a single linear reference to call SNPs and indels. (b) Gene-based linear analysis: Using orthology inference tools on annotated gene sets to determine the pan-gene repertoire and gene-level PAVs. By separating these two concepts, the manuscript can provide a more accurate and nuanced comparison, highlighting how graph-based pan-genomes aim to integrate both types of variation in a way that requires distinct workflows in a traditional linear framework.

**Answer: Thank you for this specific and helpful comment. We have reworked and updated our paragraph 5 accordingly.**

  Minor comments

  1. The authors should adopt a more authoritative tone appropriate for a standards paper. Phrases like "probably more suited" (Section 2.3) should be replaced with definitive recommendations (e.g., "we recommend the use of...") to provide clear guidance.

**Answer: Thank you for the suggestion. We rephrased the paragraph.**

  2. In Section 4.3 (Metadata requirements), the list of mandatory fields should be expanded to include the specific versions of all software and pipelines used. This is essential for ensuring full reproducibility of the analyses.

**Answer: Thanks, we have now addressed this important point and extended the paragraph to elaborate more on the importance of metadata requirements.**

  3. The case studies in Section 6 are too brief to be impactful. Each case should be slightly expanded to illustrate how the application (or lack thereof) of the proposed standards directly impacted the project's outcomes, challenges, or successes. This would provide concrete evidence for the importance of the proposed standards.

**Answer: Agreed. We updated the paragraph and extended the description of the case studies and their relevance.**

  4. Regarding the classification of genes into "core, shell, and cloud" (Section 2.3), it is crucial to state that the specific percentage thresholds used for these definitions must be explicitly reported in the metadata, as they can significantly impact downstream comparative analyses.

**Answer: We added a sentence to be more precise regarding this point.**

- Is the topic of the review discussed comprehensively in the context of the current literature?

Yes

- Are all factual statements correct and adequately supported by citations?

Yes

- Is the review written in accessible language?

Partly

- Are the conclusions drawn appropriate in the context of the current research literature?

Yes

# Reviewer report: Reviewer response for version 1

**DOI:** 10.5256/f1000research.183537.r404153

**Published:** 2025-09-03

**Authors:** Sunil Kumar Sahu

**Affiliations:** 1 State Key Laboratory of Genome and Multi-omics Technologies, BGI Research, Shenzhen, China

This article presents a very comprehensive and thoughtful set of recommendations, covering a wide spectrum from naming conventions to quality control and data sharing. I enjoyed reading it and have a few suggestions that I believe could strengthen its impact and practicality for the community.

 My main thought is that the sheer breadth of recommendations, while excellent, might feel daunting for some labs, especially those with limited resources. To make the framework more accessible, it would be incredibly helpful if the authors could more clearly distinguish between what they consider essential "minimum standards" and what are "aspirational best practices." For instance, while the framework is well-described, I found myself wishing for a more concrete, practical roadmap. The QC section, for example, lists many excellent tools (FastQC, GenomeScope, QUAST, etc.), but a visual workflow diagram would be immensely valuable. A figure illustrating the step-by-step process from raw data QC, through assembly and annotation QC, to pan-genome QC would really help readers visualize how to integrate these tools into their own standardized processes.

 Finally, on the topic of quality control, the article does a great job listing the available tools but could go further in guiding users on how to apply them. For example, some guidance on tool selection would be useful, such as which aspects of QUAST or CRAQ are best for evaluating polyploid assemblies. It would also be helpful to address how to interpret conflicting results from different tools or databases. Furthermore, in the pan-genome section, the concept of "saturation analysis" is mentioned. It would strengthen this part to include some discussion on the sample size required to confidently claim saturation, particularly for species with different ploidy levels.

 These are all meant as constructive feedback to enhance what is already a very valuable and needed framework. I hope my comments are helpful.

Is the review written in accessible language?

Yes

Are all factual statements correct and adequately supported by citations?

Yes

Are the conclusions drawn appropriate in the context of the current research literature?

Yes

Is the topic of the review discussed comprehensively in the context of the current literature?

Yes

Reviewer Expertise:

Plant genomics and evolution

I confirm that I have read this submission and believe that I have an appropriate level of expertise to confirm that it is of an acceptable scientific standard, however I have significant reservations, as outlined above.

# Response

**Published:** 2025-11-10

**Authors:** Marc Christian Heuermann

**Reviewer 1**

 Sunil Kumar Sahu ( https://orcid.org/0000-0002-4742-9870), State Key Laboratory of Genome and Multi-omics Technologies, BGI Research, Shenzhen, China

 Approved with Reservations

 This article presents a very comprehensive and thoughtful set of recommendations, covering a wide spectrum from naming conventions to quality control and data sharing. I enjoyed reading it and have a few suggestions that I believe could strengthen its impact and practicality for the community.

  My main thought is that the sheer breadth of recommendations, while excellent, might feel daunting for some labs, especially those with limited resources. To make the framework more accessible, it would be incredibly helpful if the authors could more clearly distinguish between what they consider essential "minimum standards" and what are "aspirational best practices." For instance, while the framework is well-described, I found myself wishing for a more concrete, practical roadmap. The QC section, for example, lists many excellent tools (FastQC, GenomeScope, QUAST, etc.), but a visual workflow diagram would be immensely valuable. A figure illustrating the step-by-step process from raw data QC, through assembly and annotation QC, to pan-genome QC would really help readers visualize how to integrate these tools into their own standardized processes.

**Answer: Thank you for your constructive feedback. We have added Figure 1 to the manuscript to clearly illustrate the recommended tools for each step of the pan-genome analysis workflow. The figure is now cross-referenced in both Section 3 and Section 5 for better integration with the discussed content.**

  Finally, on the topic of quality control, the article does a great job listing the available tools but could go further in guiding users on how to apply them. For example, some guidance on tool selection would be useful, such as which aspects of QUAST or CRAQ are best for evaluating polyploid assemblies. It would also be helpful to address how to interpret conflicting results from different tools or databases. Furthermore, in the pan-genome section, the concept of "saturation analysis" is mentioned. It would strengthen this part to include some discussion on the sample size required to confidently claim saturation, particularly for species with different ploidy levels.

  These are all meant as constructive feedback to enhance what is already a very valuable and needed framework. I hope my comments are helpful.

**Answer: We have revised section 3. Quality Control (QC) Standards to fully incorporate your suggestions. **

- Is the topic of the review discussed comprehensively in the context of the current literature?

Yes

- Are all factual statements correct and adequately supported by citations?

Yes

- Is the review written in accessible language?

Yes

- Are the conclusions drawn appropriate in the context of the current research literature?

Yes
